# Prevalence, incidence and characteristics of the metabolic syndrome (MetS) in a cohort of Mexican Mestizo early rheumatoid arthritis patients treated with conventional disease modifying anti-rheumatic drugs: the complex relationship between MetS and disease activity

**DOI:** 10.1186/s13075-015-0549-x

**Published:** 2015-02-20

**Authors:** Federico Parra-Salcedo, Irazú Contreras-Yáñez, Daniel Elías-López, Carlos A Aguilar-Salinas, Virginia Pascual-Ramos

**Affiliations:** Department of Rheumatology and Immunology, Instituto Nacional de Ciencias Médicas y Nutrición Salvador Zubirán, Vasco de Quiroga 15, colonia sección XVI, Tlalpan 14000, México, DF México; Department of Metabolism and Endocrinology, Instituto Nacional de Ciencias Médicas y Nutrición Salvador Zubirán, Vasco de Quiroga 15, colonia sección XVI, Tlalpan 14000, México, DF México

## Abstract

**Introduction:**

A higher prevalence of metabolic syndrome (MetS) has been described in rheumatoid arthritis (RA), along with an association with disease activity. Objectives were to describe prevalence of MetS at RA diagnosis in a cohort of Mexican Mestizo early RA patients, and to define a causal association between MetS and disease activity.

**Methods:**

The study population was a prospective cohort. At baseline and at fixed 6-months-intervals, patients had medical evaluations, fasting serum glucose, triglycerides, high-density lipoprotein cholesterol and acute reactant-phase determinations. MetS was defined according to international criteria and body mass index (BMI) ≥30 kg/m^2^ was used as a surrogate of the waist circumference. The study was approved by the internal review board. Appropriated statistics and Cox regression analysis were used. All statistical tests were two-sided and evaluated at the 0.05 significance level.

**Results:**

Up to March 2014, data from 160 patients were analyzed. At baseline, they were more frequently middle-aged females and had moderate to high disease activity. Prevalence of MetS varied from 11.3% to 17.5% in patients and was lower to that from matched controls (versus 26.3% to 30%, *P* ≤0.01).

Up to last follow-up, 39 patients (34.5%) developed incidental MetS. In the Cox regression analysis, cumulative disease activity score (DAS) 28 (odds ratio (OR): 1.81, 95% confidence interval (CI): 1.346 to 2.433, *P* = 0.000) and baseline BMI (OR: 1.13, 96% CI: 1.035 to 1.236, *P* = 0.007) were the only predictors for incidental MetS.

RA patients with incidental MetS accumulated more disease activity and had less frequent remission than their counterparts. Logistic regression analysis showed that incidental MetS (OR: 0.2, 95% CI: 0.01 to 0.99, *P* = 0.052) and baseline DAS28 (OR: 0.4, 95% CI: 0.2 to 0.9, *P* = 0.02) were the only predictors for achieving or maintaining sustained (≥6 months) remission.

**Conclusions:**

MetS prevalence in a cohort of early RA patients was lower than that from matched controls. Cumulative disease activity and higher BMI were risk factors for incidental Mets; higher baseline disease activity and incidental MetS prevented sustained remission. In addition to disease activity, MetS needs to be controlled to impact disease outcomes.

## Introduction

Cardiovascular (CV) disease is the leading cause of death in patients suffering from rheumatoid arthritis (RA) [1-3]. Factors that contribute to increased CV risk are genetic factors, chronic inflammation, traditional CV risk factors along with disease and treatment-related factors [4-11]. More recently, it has been proposed that metabolic syndrome (MetS) should be added to the list; in the general population, MetS identifies patients at risk of developing coronary heart disease later in life, with a relative risk of 2.7 [12].

MetS refers to a clustering of specific classical CV risk factors, the combination of which is thought to be associated with increased CV risk beyond the sum of the individual components [13]. The existing literature suggests that inflammatory processes are involved in MetS pathogenesis. Regarding prevalence of MetS in RA, there have been conflicting results but the most recent meta-analysis of 12 observational, cross-sectional studies involving 2,283 Caucasian and Asian RA patients showed a significantly increased prevalence when compared with 4,403 controls [14]. Chung and colleagues found that the frequency of MetS was greater in patients with long-term disease (42%) than in patients with early arthritis (30%) [15]; Dao and colleagues confirmed the association between RA and MetS in 105 Vietnamese women with short disease duration and additionally showed that individual components of the MetS, such as hypertension, high-density lipoprotein cholesterol levels and abdominal obesity, were more frequent in RA patients than in their matched controls [16]. Additional studies have found association with some components of the MetS and disease activity [17-19], although the cross-sectional design of the studies prevents identification of causal associations.

In México, data from the National Health and Nutrition Survey 2006 have shown a high prevalence of MetS among adults, ranging from 36.8 to 49.8% depending on the criteria applied. Notably, MetS was more prevalent among women, among the less educated individuals and among those with lower incomes [20]. Hispanic population-based studies have also shown an increased susceptibility in Mexican adults and confirmed local data [21].

A goal of clinical management in individuals with MetS is to reduce the risk of clinical atherosclerotic disease (and related comorbid conditions), which is a major cause of death in patients with RA [22]. Accordingly, RA patients with such comorbidity need to be identified. Furthermore, the relationship between disease activity and MetS needs to be clarified in order to understand how achievement of major outcomes as sustained remission may further impact on CV mortality. In the literature there is a paucity of data in Latin American countries where both entities, MetS and RA, have distinctive epidemiological, serological and clinical characteristics [23].

The main objectives of the study were: first, to describe the prevalence and characteristics of MetS at RA diagnosis in a cohort of Mexican Mestizo early RA patients and compare these with data from healthy matched controls; and second, to define the relationship between MetS and disease activity. Based on our cohort’s characteristics (high disease activity at enrollment, high remission rate after treat-to-target strategy and substantial follow-up [24]), two secondary objectives were established: to investigate MetS as a risk factor to achieve a first sustained remission status; and to determine whether incidental MetS over follow-up impacts disease activity-related outcomes.

## Methods

### Setting and study population

The Early Arthritis Clinic of the Instituto Nacional de Ciencias Médicas y Nutrición Salvador Zubirán was established in February 2004. Patients referred to the clinic had suspicion of RA and symptom duration of less than 12 months. Patients with confirmed recent-onset RA also attend the clinic; in such patients, the time from diagnosis to first evaluation is ≤5 weeks.

Patients received treat-to-target-oriented treatment. Traditional disease-modifying anti-rheumatic drugs (DMARDs) were used in 99% of our population, with or without corticosteroids (20 to 40%).

### Clinical evaluations

At baseline, a complete medical history and sociodemographic characteristics were obtained. Blood samples after 9 to 12 hours fast included at least serum glucose (GLU), total cholesterol, triglycerides (TG), high-density lipoprotein-cholesterol (C-HDL), low-density lipoprotein-cholesterol, uric acid, erythrocyte sedimentation rate (ESR), C-reactive protein (CRP), rheumatoid factor and antibodies to cyclic citrullinated proteins (ACCP). Urine analysis was also performed. For serum GLU and lipid determinations, commercially available reagents from Beckman Co. (Limerick, Ireland) for the Synchron analyzer (Laboratorios Clínicos del Instituto Nacional de Ciencias Médicas y Nutrición Salvador Zubirán, Mexico City, Distrito Federal, México) were used. The laboratory obtained ISO 9001:2008 certification (since 30 April 2004, valid from 29 April 2013 until 28 April 2016).

Standard baseline and consecutive rheumatic evaluations included, height, weight and blood pressure measurements, extended joint counts, physician and patient-reported outcomes [25,26], disease activity score evaluated in 28 joints (DAS28) [27], adverse events and comorbidity. Blood pressure was measured twice in each case in which initial measurement was ≥130/90 mmHg unless the patient had a previous diagnosis of systemic arterial hypertension. Measurements were performed after a 5-minute resting period. Body weight and height were performed by a trained nurse, usually on the same equipment that was regularly calibrated according to the manufacturer’s recommendations.

Patients were evaluated every 2 months during the first 2 years of follow-up, and thereafter every 2, 4 or 6 months (fixed and scheduled for all patients). At fixed 6-month intervals, analysis in order to measure serum GLU, total cholesterol, TG, C-HDL, low-density lipoprotein-cholesterol, uric acid, ESR, CRP and urine analysis were scheduled. Medical evaluations were performed by the same rheumatologist and the information was recorded on standardized formats.

RA treatment records included previous treatment (defined as prescribed during the month prior to the baseline evaluation) and current treatment (at baseline and consecutive evaluations), which included names, doses, schedule, order start and stop dates of DMARDs, of corticosteroids and of nonsteroid anti-inflammatory drugs. In addition, any other drug indicated for a comorbid condition was also recorded; for the present study, names, doses, schedule, order start and stop dates of antihypertensive, antidiabetic and lipid-lowering medications were obtained. Information regarding treatment was recorded after a direct interview on a drug record registry applied at scheduled evaluations [28].

Up to March 2014, charts from 162 consecutive patients with early RA were reviewed. In order to accomplish the first objective, only RA patients with completed baseline blood pressure, weight and height, and serum TG, C-HDL and GLU levels were included (two patients were excluded); there were 160 patients left.

Of the 160 patients with complete baseline data, 19 were lost to follow-up soon after the baseline evaluation (and their baseline characteristics did not differ from those patients with follow-up) and 28 additional patients had prevalent MetS. Accordingly, there were 113 patients with at least 6 months of follow-up and MetS-free baseline, in whom incidental MetS was investigated.

Finally, to accomplish the second objective related to MetS and its impact on remission, data from patients with at least 24 months of follow-up were analyzed (*n* = 133). This lag time was chosen based on previous reports that defined median time to sustained remission in our cohort as 13.6 (±8.8) months [29].

### Control population

To achieve the first objective, one control for each RA patient was randomly selected from a local database that included data from more than 10,000 Mexican Mestizo adults without either known medical condition or treatment, in whom serum samples were obtained in order to investigate the prevalence of the MetS in Central Mexico. Controls were matched according to age (±5 years), gender, smoking habit (see definition below), years of education, menopause status and urban residency.

To achieve the second objective, a case–control study nested within a cohort was designed; cases consisted of RA patients with incidental MetS and controls consisted of RA patients who never developed MetS during their follow-up. Controls were matched according to gender, age, index date (as defined when MetS was diagnosed or equivalent visit in MetS-free RA patients), menopause status, presence of rheumatoid factor or ACCP, and follow-up.

### Variables and definitions

Body mass index (BMI) was calculated as weight (kg)/height (m^2^).

Smoking status was self-reported at the baseline evaluation and categorized as never smoker, former smoker (at least one cigarette per day for at least 3 months during their lifetime but who do not currently smoke) or current smoker (at least one cigarette/day for at least 3 months).

Postmenopausal women were self-reported and defined as women ≥48 years of age who have been amenorrheic for at least 2 years, or females with bilateral oophorectomy or females with documented hysterectomy aged ≥47 years [30].

Fasting GLU, uric acid, total cholesterol, low-density lipoprotein-cholesterol, C-HDL and TG were measured in serum and reported in milligrams per deciliter.

Sustained remission was defined based on the DAS28 if score ≤2.6 [31] and according to 2012 American College of Rheumatology/European League Against Rheumatism criteria [32] and if maintained for at least 6 months of follow-up.

Hypertension was defined if recorded on the charts, or antihypertensive medication was used, or a diastolic blood pressure ≥90 mmHg was detected or a systolic blood pressure ≥140 mmHg was detected.

Diabetes mellitus *was* defined if a physician diagnosis was recorded on the charts, if antidiabetic medication was recorded or if a fasting blood sugar level ≥126 mg/dl was detected.

MetS was defined according to three sets of criteria (Table 1) [22,33,34]. In all of these sets, BMI ≥30 kg/m^2^ was considered a surrogate of waist circumference ≥102 cm in males and ≥88 cm in females.

**Table 1:** **Metabolic syndrome sets of criteria**

**2001 NCEP ATP III definition**	**AHA/NHLBI definition**	**IDF definition**
**(any three of five)**	**(any three of five)**	**(required** ^**a**^ **and any two of four criteria left)**
BMI ≥30 kg/m^2^	BMI ≥ 30 kg/m^2^	BMI ≥30 kg/m^2 a^
TG ≥150 mg/dl	TG ≥150 mg/dl or treatment for elevated TG	TG ≥150 mg/dl or treatment for elevated TG
C-HDL <40 mg/dl in men	C-HDL <40 mg/dl in men	C-HDL <40 mg/dl in men
C-HDL <50 mg/dl in women	C-HDL <50 mg/dl in women	C-HDL <50 mg/dl in women
Blood pressure ≥130/85 mmHg or previous hypertension diagnosis	Blood pressure ≥130/85 mmHg or previous hypertension diagnosis	Blood pressure ≥130/85 mmHg or previous hypertension diagnosis
Fasting glucose ≥110 mg/dl or previous type 2 DM	Fasting glucose ≥100 mg/dl or previous type 2 DM	Fasting glucose ≥100 mg/dl or previous type 2 DM

Criteria according to the National Cholesterol Education Program Adult Treatment Panel III (NCEP ATP III) definition [33], the American Heart Association/National Heart, Lung and Blood Institute (AHA/NHLBI) definition [22] and the International Diabetes Federation (IDF) definition [34]. BMI, body mass index; C-HDL, high-density lipoprotein-cholesterol; DM, diabetes mellitus; TG, triglycerides.

^a^Is a prerequisite risk factor for the MetS diagnosis.

### Ethics

The study was approved by the Ethics Committee of the Instituto Nacional de Ciencias Médicas y Nutrición. Written informed consent was obtained in order to have the patient’s charts reviewed and data presented in scientific forums or published.

### Statistical analysis

Distribution of each variable was analyzed. Student’s *t* test and the chi-square test were used for normally distributed variables and the Mann–Whitney *U* test for non-normally distributed variables.

To summarize cumulative outcomes (disease activity, disability) or variables (CRP, ESR), the mean of consecutive values from corresponding evaluations was obtained.

Follow-up missing data varied from 3% (for BMI) to 20% (for serum GLU levels). Imputation was calculated for linear regression method, considering an arbitrary pattern of missing values.

Prevalence of MetS was determined based on different sets of criteria applied at baseline or within 2 months from baseline evaluation. The prevalence of the MetS was compared between early RA and matched controls using Fisher’s exact test.

Logistic and Cox regression’s models were used: to evaluate the risk of MetS (defined at the baseline evaluation); to achieve remission; to identify predictors of incidental MetS; and to investigate incidental MetS as a predictor of sustained remission. In all cases, selection of variables to be included was based on their statistical significance in the bivariate analysis; a cutoff point was established based on the number of variables *a priori* included in order to avoid overfitting of the models; based on the number of outcomes of interest, four or five variables were included. Correlation between variables was also examined, and when significant only one variable was selected. Selection was based on variables’ clinical relevance; in all cases, correlated variables were switched into the models to test the integrity of the results. Finally, if a variable was deemed to be clinical significant, its inclusion was forced into the model (cumulative treatment).

In the first model, there were no variables with statistical significance in the univariate analysis and the covariates included *a priori* were age, gender and MetS at baseline evaluation.

In the second analysis, variables selected were age (correlated to menopause, *r* = 0.5 and to years of formal education, *r* = 0.3), BMI, cumulative DAS28 (correlated to cumulative ESR, *r* = 0.75, to cumulative CRP, *r* = 0.4 and to cumulative HAQ, *r* = 0.67) and follow-up (correlated to erosions at index date, *r* = 0.26), *P* ≤0.001 for all correlations.

In the third analysis, variables included were baseline DAS28 (correlated to ESR, *r* = 0.6, to HAQ, *r* = 0.67 and to CRP, *r* = 0.6), incidental MetS (correlated to BMI, *r* = 0.4 and to obesity, *r* = 0.3), disease duration at baseline, ACCP and age (*P* ≤0.001 for correlations); cumulative treatment was also forced into models.

All statistical tests were two-sided and evaluated at the 0.05 significance level. Statistical analysis was performed using the SPSS/PC program (v.17.0; IBM SPSS Statistics, Chicago, IL, USA).

## Results

### Population characteristics at cohort entry

At baseline 160 patients had complete data, as summarized in Table 2. Patients were more frequently middle-aged females (142 female, mean ± standard deviation age: 38.1 ± 12.8 years) and had median (range) BMI of 25.9 kg/m^2^ (18.1 to 46.8 kg/m^2^). The population had short disease duration, was most frequently rheumatoid factor-positive and ACCP-positive, and had high disease activity and moderate disability. A minority of patients had erosive disease (9.4%). Comorbid conditions were present in 82 patients (51.3%), among which were obesity in 35 patients (21.9%), hypertension in 13 patients (8.1%) and diabetes in six patients (3.8%). Few patients were receiving DMARDs when referred to the clinic (27.5%), although 36.9% were on oral corticosteroids.

**Table 2:** **Baseline characteristics of the whole population and comparison between patients with/without metabolic syndrome (according to AHA/NHLBI definition)**

**Characteristic**	**Controls**	**Whole population**	**Patients with MetS**	**Patients without MetS**	***P*** **value** ^**a**^
	**(** ***n*** **= 160)**	**(** ***n*** **= 160)**	**(** ***n*** **= 28)**	**(** ***n*** **= 132)**	
**Socio-demographic**					
Female gender	142 (88.8)	142 (88.8)	22 (78.6)	120 (90.9)	0.09
Age (years)	38 ± 12.4	38.1 ± 12.8	43.1 ± 10.5	37 ± 13	0.02
Years of formal education	10.9 ± 3.8	10.9 ± 3.8	10.8 ± 3.5	11 ± 3.8	0.87
Current smokers	16 (10)	16 (10)	2 (7.1)	14 (10.6)	0.74
Females with menopause (142 female)	14 (9.9)	14 (9.9)	3 (13.6)	11 (9.2)	0.46
BMI (kg/m^2^)	25.5 (19.2 to 41)	25.9 (18.1 to 46.8)	30.2 (23 to 46.8)	24.8 (18 to 41.7)	0.000
**Disease characteristics**					
Disease duration (months)	NA	5.3 (0.47 to 11.5)	4.3 (1.4 to 10.5)	5.3 (0.5 to 12.5)	0.13
Patients with RF	NA	130 (81.3)	25 (89.3)	105 (79.5)	0.29
Patients with ACCP	NA	134 (83.8)	26 (92.9)	108 (81.8)	0.26
DAS28	NA	6 (2 to 8.7)	6.1 (3 to 8.6)	6 (2 to 8.7)	0.94
ESR (mm/hour)	NA	23 (2 to 102)	22 (3 to 77)	23 (2 to 102)	0.86
CRP (mg/dl)	NA	0.73 (0 to 14.7)	0.76 (0.1 to 8.5)	0.73 (0 to 14.7)	0.95
HAQ	NA	1.4 (0 to 3)	1.4 (0 to 3)	1.5 (0.3)	0.39
Patients with erosions	NA	15 (9.4)	6 (21.4)	9 (6.8)	0.03
**Comorbid conditions**					
Patients with ≥1 comorbid condition	NA	82 (51.3)	22 (78.6)	60 (45.5)	0.001
Patients with diabetes	NA	6 (3.8)	5 (17.9)	1 (0.8)	0.001
Patients with hypertension	NA	13 (8.1)	9 (32.1)	4 (3)	0.000
Patients with BMI ≥30 kg/m^2^	NA	35 (21.9)	18 (75)	17 (12.5)	0.000
**Treatment**					
Patients with DMARDs	NA	44 (27.5)	9 (32.1)	35 (26.5)	0.64
Patients with corticosteroids	NA	59 (36.9)	11 (39.3)	48 (36.4)	0.83
Patients with antimalarials	NA	26 (16.3)	5 (17.9)	21 (15.9)	0.78
Patients with other drugs	NA	83 (51.9)	14 (50)	69 (52.3)	0.84

Data presented as *n* (%), mean ± standard deviation or median (range). ACCP, antibodies to cyclic citrullinated peptides; AHA/NHLBI, American Heart Association/National Heart, Lung and Blood Institute; BMI, body mass index; CRP, C-reactive protein; DAS28, disease activity score evaluated in 28 joints; DMARD, disease-modifying anti-rheumatic drug; ESR, erythrocyte sedimentation rate; HAQ, Health Assessment Questionnaire; MetS, metabolic syndrome; NA, not applicable; RF, rheumatoid factor. ^a^Comparison between rheumatoid arthritis patients with/without MetS.

### Characteristics and prevalence of metabolic syndrome in RA patients and comparison with matched healthy controls (first objective)

**Figure 1:**
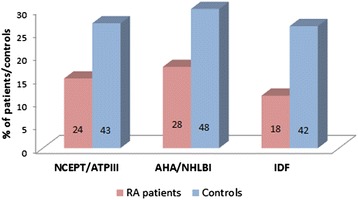
**Comparison of prevalence of metabolic syndrome between rheumatoid arthritis patients and matched controls.** Numbers inside bars represent the number of patients and controls with metabolic syndrome according to the three different definitions. AHA/NHLBI, American Heart Association/National Heart, Lung and Blood Institute; IDF, International Diabetes Federation; NCEPT/ATPIII, National Cholesterol Education Program Adult Treatment Panel III; RA, rheumatoid arthritis.

In RA patients, the prevalence of MetS varied from 11.3% (according to the International Diabetes Federation definition) to 17.5% (according to the American Heart Association/National Heart, Lung and Blood Institute (AHA/NHLBI) definition). In healthy matched controls, prevalence of Mets was significantly higher and varied from 26.3% (according to the International Diabetes Federation definition) to 30% (according to the AHA/NHLBI definition), as shown in Figure 1 (*P* ≤0.01 for all comparisons).

Distribution of individual components of the MetS in RA patients and controls was as follows: BMI ≥30 kg/m^2^ ranged from 64.3 to 100%, TG ≥150 mg/dl from 92.9 to 95.8%, C-HDL <40 mg/dl (men) or <50 mg/dl (women) from 94.4 to 96.4%, blood pressure ≥130/85 mmHg (or previous hypertension diagnosis) from 20.8 to 22.2%, and fasting GLU ≥100 mg/dl (or previous type 2 diabetes mellitus) from 27.8 to 53.6%. Comparison of individual components between RA patients and healthy controls showed that the former less frequently had the BMI component and tend to more frequently have the GLU component, as shown in Table 3.

**Table 3:** **Comparison of the distribution of individual components of metabolic syndrome (according to AHA/NHLBI definition) between RA patients and controls**

	**Early RA patients ** **(** ***n*** **= 160)**	**Controls** **(** ***n*** **= 160)**	***P*** **value**
Patients who met AHA/NHLBI MetS definition	28 (17.5)	48 (30)	0.01
Patients within each category of MetS definition with a particular individual component			
BMI component	18 (64.3)	42 (87.5)	0.02
TG component	26 (92.9)	43 (89.6)	1
C-HDL component	27 (96.4)	46 (95.8)	1
Blood pressure component	10 (35.7)	11 (22.9)	0.29
Fasting glucose component	15 (53.6)	16 (33.3)	0.10

Data presented as *n* (%). AHA/NHLBI, The American Heart Association/National Heart, Lung and Blood Institute; BMI, body mass index; C-HDL, high-density lipoprotein-cholesterol; MetS, metabolic syndrome; RA, rheumatoid arthritis; TG, triglycerides.

Comparison of baseline characteristics between patients with/without prevalent MetS is summarized in Table 2. Patients with MetS according to AHA/NHLBI definition were older, had greater BMI, had more erosive disease and tended to be more frequently men than their counterparts. As expected, patients with MetS had also more diabetes, hypertension and obesity, while other comorbid conditions were equally represented. Variables related to disease activity and treatment were similar in both groups. Similar results were obtained when the 2001 National Cholesterol Education Program Adult Treatment Panel III definition and the International Diabetes Federation definition were applied (data not shown).

### Metabolic syndrome and the probability for achieving remission status (second objective)

As already mentioned, 133 patients had at least 24 months of follow-up, of whom 108 (81%) had at least ≥1 sustained American College of Rheumatology/European League Against Rheumatism remission state. They achieved remission at (median, range) 14 months (4 to 80 months) of follow-up and remained in remission for 16 months (6 to 104 months). Twenty-two out the 108 patients who achieved remission (20.4%) had MetS according to AHA/NHLBI criteria and no differences were seen in the percentage of patients achieving remission, time to remission state and time on sustained remission between patients with/without MetS. Cox regression analysis did not identify MetS as a risk factor for any construct related to remission (data not shown). Similar results were obtained when remission was defined according to DAS28 (data not shown).

### Incidental metabolic syndrome in RA patients and impacts on major outcomes (second objective)

**Figure 2:**
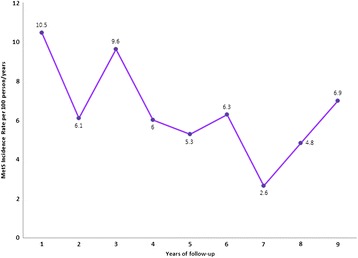
**Annual metabolic syndrome incidence rate.** MetS, metabolic syndrome.

To explore the relationship between MetS and disease activity-related outcomes we first identified RA patients who developed incidental MetS at some point during their follow-up. According to the AHA/NHLBI definition, 39 patients (34.5%) out of 113 baseline MetS-free patients with adequate follow-up developed incidental MetS. The global MetS incidence rate was 8 per 100 persons/year; as shown in Figure 2, the annual incidental rate of MetS tended to decrease after the third year of follow-up.

Baseline characteristics and cumulative disease characteristics up to the index date (when incident MetS was identified/or up to last follow-up) were then compared between RA patients with/without incidental Mets. Results are summarized in Table 4 and showed that RA patients who developed incidental MetS were older, were less educated, were more frequently postmenopausal, had higher BMI, had more cumulative disease activity and disability, had previous incidental MetS diagnosis, developed erosive disease more frequently than their counterparts and had longer follow-up. In order to identify predictors of MetS, different Cox regression models were tested in which baseline demographic and anthropometric variables were included in addition to cumulative variables related to disease activity. Table 5 presents the different models tested; in all of the models, cumulative DAS28 and baseline BMI were the only predictors for incidental MetS. When RA-related treatment was forced into the model, similar results were obtained.

**Table 4:** **Comparison of rheumatoid arthritis patient characteristics between patients with/without incidental metabolic syndrome**

**Characteristics**	**MetS-free patients**	**Patients with incidental MetS**	***P*** **value**
	**(** ***n*** **= 74)**	**(** ***n*** **= 39)**	
**Socio-demographic**			
Female gender	68 (91.9)	35 (89.7)	0.74
Age (years)	33.6 ± 11.9	42.7 ± 13.8	0.000
Years of formal education	11.6 ± 3.6	10 ± 4.2	0.04
Current smokers	10 (13.5)	2 (5.1)	0.21
Females with menopause (142 female)	4 (4.4)	7 (20)	0.03
BMI (kg/m^2^)	23.9 (21.5 to 26.6)	26.8 (24.5 to 29.8)	0.000
**Disease characteristics**			
Disease duration (months)	5.4 (3.9 to 7)	5.3 (2.9 to 7.3)	0.38
Patients with RF	59 (79.7)	29 (74.4)	0.63
Patients with ACCP	63 (85.1)	29 (74.4)	0.21
Cumulative^a^ DAS28	2.4 (2.1 to 3.05)	3.3 (2.7 to 5.8)	0.000
Cumulative^a^ ESR (mm/hour)	12.5 (8.6 to 18.2)	21 (10.2 to 35)	0.008
Cumulative^a^ CRP (mg/dl)	0.28 (0.15 to 0.58)	0.52 (0.26 to 1.21)	0.007
Cumulative^a^ HAQ	0.2 (0.12 to 0.43)	0.38 (0.25 to 1.13)	0.001
Patients with erosions at index date^a^	27 (34.5)	23 (59)	0.03
Follow-up (months)	72 (42 to 94.5)	90 (70 to 108)	0.003
**Comorbid conditions**			
Patients with ≥1 comorbid condition	35 (47.3)	20 (51.3)	0.67
Patients with diabetes	0	1 (2.6)	0.35
Patients with hypertension	1 (1.4)	3 (7.7)	0.12
Patients with BMI ≥30 kg/m^2^	3 (4.1)	10 (25.6)	0.001
**Treatment at baseline**			
Patients with DMARDs	15 (32.6)	15 (38.5)	0.12
Patients with corticosteroids	26 (35.1)	12 (30.8)	0.68
Patients with antimalarials	12 (16.2)	8 (20.5)	0.61
Patients with other drugs	37 (50)	22 (56.4)	0.56

Data presented as *n* (%), mean ± standard deviation or median (range). ACCP, antibodies to cyclic citrullinated peptides; BMI, body mass index; CRP, C-reactive protein; DAS28, disease activity score evaluated in 28 joints; DMARD, disease-modifying anti-rheumatic drug; ESR, erythrocyte sedimentation rate; HAQ, Health Assessment Questionnaire; MetS, metabolic syndrome; RF, rheumatoid factor. ^a^Considered up to MetS diagnosis in patients with incidental MetS and up to last follow-up in the patients without incidental MetS.

**Table 5:** **Cox regression models to define predictors for incidental metabolic syndrome (according to AHA/NHLBI definition)**

	**Model 1**	**Model 2**	**Model 3**
Age	1.005, 0.98 to 1.03	1.009, 0.98 to 1.04	0.99, 0.98 to 1.02
BMI	1.14, 1.04 to 1.26		1.14, 1.04 to 1.25
BMI ≥25 kg/m^2^		3.07, 1.7 to 9.26	
Cumulative DAS28	1.79, 1.33 to 2.43	1.92, 1.41 to 2.62	1.91, 1.37 to 2.68
Erosions at index date			0.71, 0.32 to 1.6
Follow-up	0.99, 0.97 to 1.004	0.99, 0.98 to 1.01	

Data presented as Exp (β), 95% confidence interval. AHA/NHLBI, American Heart Association/National Heart, Lung and Blood Institute; BMI, body mass index; DAS28, disease activity score evaluated in 28 joints.

**Figure 3:**
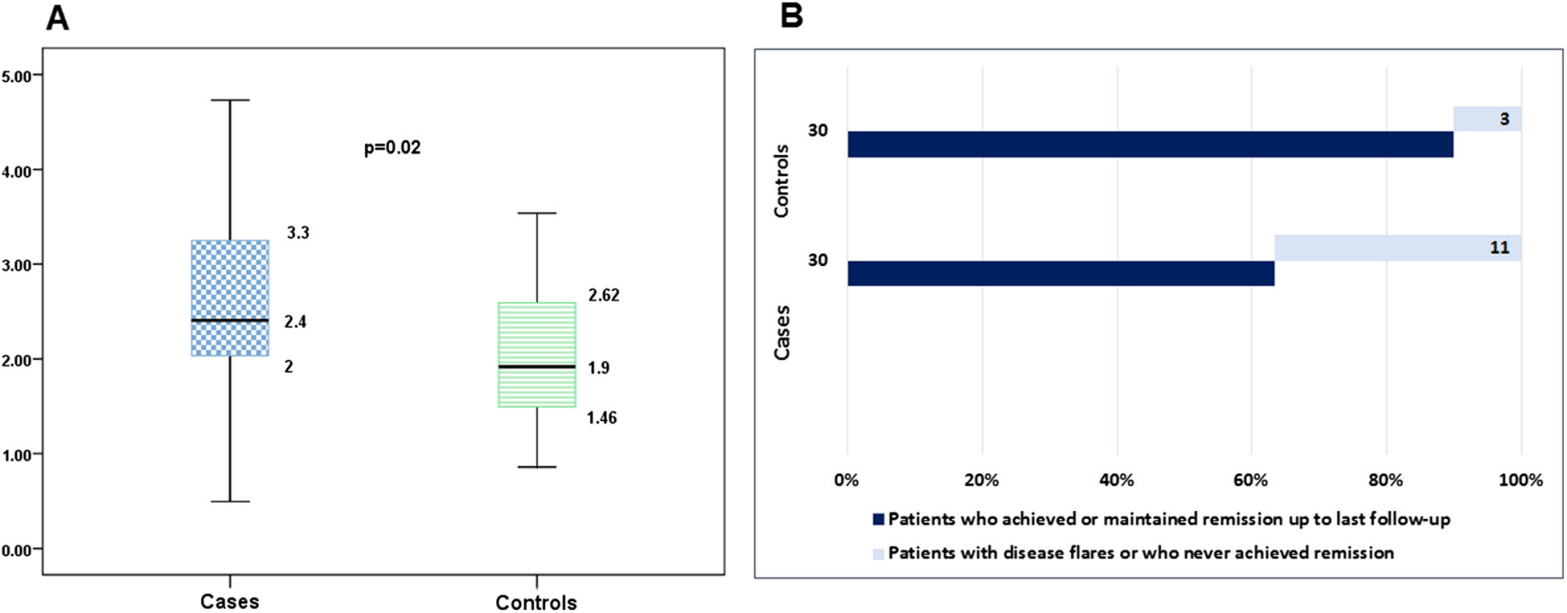
**Comparison of disease activity-related outcomes after the index date between cases and controls.** Comparison of disease activity-related outcomes after the index date, between rheumatoid arthritis (RA) patients with incidental metabolic syndrome (Mets; cases) and their matched controls (RA patients who did not develop incidental MetS up to last follow-up). **(A)** Median (range) cumulative disease activity score evaluated in 28 joints after the index date in RA patients who developed incidental MetS and their controls. **(B)** Distribution (*n* (%)) of patients and controls who achieved or maintained remission and of patients and controls with disease flare or who never achieved remission, from the index date up to last follow-up.

We then explored the impact of MetS on disease activity. A case–control study nested within a cohort was designed; among the 39 RA patients with incidental MetS, 30 were paired to 30 corresponding matched controls (age, gender, index date, menopause status, RF, ACCP and follow-up). Cumulative outcomes related to disease activity were compared between RA cases and controls after the index date and results are summarized in Figure 3A,B. RA patients with incidental MetS had higher median (range) cumulative DAS28 after the index date than RA patients who never developed MetS over follow-up (Figure 3A); also, less RA patients from the former group achieved or maintained remission while more patients had disease flare or never achieved remission when compared with the control group (Figure 3B). We then compared characteristics from patients who achieved or maintain remission after the index date (*n* = 46) and those who never achieved remission (*n* = 14); as shown in Table 6, the former had lower BMI, longer disease duration at baseline evaluation, lower baseline disease activity (as per DAS28, ESR and CRP) and disability, developed incidental MetS less frequently and were more frequently obese at the baseline evaluation. Baseline (not shown) and cumulative treatment before sustained remission was similar (corticosteroid use and number of DMARDs/patient). Finally, logistic regression analysis was performed in order to identify predictors for remission after incident MetS. Table 7 presents the different models tested; in all of them, incidental MetS and baseline DAS28 were the only predictors for achieving or maintaining a sustained remission status.

**Table 6:** **Comparison of characteristics between RA patients who never achieved remission and those who achieved and maintained remission (case–control study, nested within the cohort)**

**Characteristic**	**RA patients who achieved and maintained remission**	**RA patients who never achieved sustained remission**	***P*** **value**
	**(** ***n*** **= 46)**	**(** ***n*** **= 14)**	
**Socio-demographic**			
Female gender	40 (87%)	14 (100)	0.32
Age (years)	38.2 ± 10.5	38.7 ± 12.5	0.88
Years of formal education	10.5 ± 8.8	10 ± 7.7	0.43
Current smokers	5 (10.9)	0 (0)	0.33
Females with menopause (142 female)	2 (5)	1 (7)	1
Baseline BMI (kg/m^2^)	25.1 (22 to 27.5)	27.8 (23.1 to 30.4)	0.05
**Disease characteristics**			
Disease duration (months)	5.8 (4.1 to 7.7)	3.3 (1.6 to 6)	0.03
Patients with ACCP	38 (82.6)	13 (92.9)	0.67
Baseline DAS28	6 (4.6 to 6.8)	7.3 (6.5 to 7.8)	0.002
Baseline ESR (mm/hour)	24.5 (14.3 to 37)	43 (28.3 to 59.5)	0.012
Baseline CRP (mg/dl)	1 (0.3 to 3.4)	2.4 (1.4 to 6.1)	0.05
Baseline HAQ	1.4 (0.6 to 2)	2.3 (1.6 to 3)	0.008
Patients with erosions at baseline	5 (11)	0 (0)	0.33
Follow-up (months)	88 (58.5 to 108)	87 (57 to 108)	0.96
**Incidental MetS**	19 (41.3)	11 (78.6)	0.03
**Comorbid conditions**			
Patients with diabetes	1 (2.2)	0 (0)	1
Patients with hypertension	1 (2.2)	0 (0)	1
Patients with BMI ≥30 kg/m^2^	4 (8.7)	6 (42.9)	0.007
**Treatment up to sustained remission** ^**a**^			
Number of DMARDs/patient	2 (2 to 2.5)	2.5 (2 to 2.75)	0.08
Patients with corticosteroids	30 (65.2)	9 (64.3)	1

Data presented as *n* (%), mean ± standard deviation or median (range). ACCP, antibodies to cyclic citrullinated peptides; BMI, body mass index; CRP, C-reactive protein; DAS28, disease activity score evaluated in 28 joints; DMARD, disease-modifying anti-rheumatic drug; ESR, erythrocyte sedimentation rate; HAQ, Health Assessment Questionnaire; MetS, metabolic syndrome; RA, rheumatoid arthritis. ^a^In those who never achieved remission, complete follow-up was considered.

**Table 7:** **Regression models to define predictors for sustained remission**

	**Model 1 (parsimonious)**	**Model 2**	**Model 3**
DAS28 at baseline	0.44, 023 to 0.85	0.50, 0.21 to 0.85	0.42, 0.21 to 0.85
Incidental MetS	0.23, 0.05 to 1.00	0.18, 0.04 to 0.89	0.23, 0.05 to 1.09
Disease duration at baseline		1.28, 0.94 to 1.74	1.26, 0.93 to 1.70
ACCP		0.20, 0.02 to 2.29	
Age			0.97, 0.90 to 1.04

Data presented as Exp (β), 95% confidence interval. ACCP, antibodies to cyclic citrullinated peptides; DAS28, disease activity score evaluated in 28 joints; MetS, metabolic syndrome.

## Discussion

In the present study we found that 11 to 18% of Mexican Mestizo early RA patients had MetS diagnosed as a comorbid condition and this figure was lower for prevalence of MetS in healthy controls. Distribution of the TG and C-HDL components of MetS was similar between patients and controls, but early RA patients presented less frequently the BMI component while the controls showed a similar tendency for the GLU and hypertension components. Patients with prevalent MetS were older, had greater BMI, had more radiographic damage and more frequently had comorbid conditions related to MetS than RA patients without the syndrome.

In 2008, Chung and colleagues were the first to demonstrate that prevalence of MetS in patients with RA was increased when compared with matched controls [15]. Patients with long-standing disease had the greatest prevalence. A recent meta-analysis by Zhang and colleagues has proven further evidence in support of a higher prevalence of MetS in RA patients, with an overall odds ratio of 1.24 (95% confidence interval, 1.03 to 1.50), although geographic area and the criteria used influenced the association [14]; interestingly, none of the 12 studies included in the meta-analysis was performed in Mexican patients. Conflicting results regarding this topic were highlighted in two excellent reviews by da Cunha and colleagues in 2011 [35] and Ferraz-Amaro and colleagues in 2013 [36]; both studies concluded that MetS is not uncommon in patients with RA but mentioned published exceptions with regard to a greater prevalence in RA patients [18,37-40]. More recently, Mok and colleagues estimated a prevalence of 20% in 699 Chinese patients, of whom 209 had disease duration of less than 2 years [41]. In most of the studies reported (including exceptions), matching of controls has been restricted to age and sex, and sometimes to race. We performed a more precise matching, including characteristics that do affect the prevalence of MetS in Mexican Mestizo patients such as years of education [20], in addition to age, gender, smoking habit, menopause status and urban residency. This unique characteristic to our study, along with a population with short disease duration and younger age at diagnosis (mean age 38.1 years), may explain our prevalence figure of MetS in RA patients in the lower range (up to 18%), which in fact is similar to that reported by Zonana-Nacach and colleagues in the only study performed in Mexican patients [42]. Also, our prevalence figure of MetS in matched controls, which may be considered high, is similar to that reported in the National Health and Nutrition Survey in 2006 [20], when analyzed by age group.

We found among the individual components of MetS that differences between patients and controls were observed for the BMI component, which was more frequent in controls; meanwhile, opposite tendencies were seen for the hypertension and GLU components, partially consistent with studies performed in early RA [15,16]. More than one-half of our active RA patients with MetS presented the GLU component; this figure may be explained by a negative correlation of high-grade inflammation with circulating adiponectin concentrations as proposed by González-Gay and colleagues; their study involved patients with severe RA in whom (low) adiponectin concentrations further correlated with high plasma GLU [43]. Ultimately, discrepancies with other studies may be explained by the particular distribution of individual components of the MetS in our population along with distinctive epidemiological, clinical and serological characteristics of Mexican patients with RA.

Our early arthritis cohort of Mexican Mestizo patients has particular characteristics [24,28,29] that were considered for the second aim of the study and confer relevance to findings, such as a homogeneous population of patients with early disease and substantial comorbidity (real-life patients), standardized and complete follow-up by the same rheumatologist in a real clinical setting, periodic evaluation of comorbidity, treatment and physician-reported and patient-reported outcomes, use of conventional DMARDs according to a treat-to-target strategy, and substantial follow-up. To our knowledge, this is the only study in which incident MetS is determined so cause–effect inferences on the relationship between MetS and disease activity were possible. We first described the MetS incidence rate as 8 per 100 persons/year and showed that the annual MetS incidence rate decreased after the third year; this can be explained by a greater burden of disease activity at RA diagnosis and/or by a more intensive search of comorbid conditions in patients first attending a clinic. To our knowledge there is no information available in the literature that may help us to contrast these data.

We could not find a relation between prevalent MetS and the different outcomes related to remission (as time to remission and time on sustained remission). Similar to results described in some studies, prevalent MetS was not associated with disease activity parameters [37,38] but with erosive disease, although the latter is generally linked to a greater disease activity burden. Nonetheless, the most relevant findings were that incidental MetS and baseline disease activity were predictors for achieving and maintaining a remission status. Also, looking at the problem from the other side of the coin, cumulative disease activity and BMI were both predictors of incidental MetS. Although there are published studies that have established an association between MetS and disease-related outcomes [14,16-19,37,38,44-47], data based on a prospective design that allows risk evaluations are very limited. Gremese and Ferraccioli in their review described unpublished data from 115 long-standing RA patients with disease activity and starting anti-TNF; RA patients carrying MetS had lower chance to achieve a good response [48]. Our data support that MetS has an inflammatory milieu favoring the occurrence of RA or leading to a more severe disease. On the contrary, MetS contributes to a poorer response to therapy in RA patients, probably due to the additive effect of its low-grade inflammation to the great inflammatory burden of RA. The most relevant point from these observations may be related to the complex relationship of the different pathways, cytokines and adipocyte-derived mediators driving inflammation in RA patients with MetS; as an example, proinflammatory cytokines such as tumor necrosis factor alpha are involved in the pathogenesis of insulin resistance while resistin (an adipocyte-derived mediator) also plays an important role in the inflammatory cascade and undergoes a dramatic reduction after anti-tumor necrosis factor alpha therapy [49]. In fact, the benefits of tumor necrosis factor alpha blockade in RA patients go far beyond the local (synovial) inflammatory process, and extend to other biological actions such as improvement of endothelial dysfunction and of insulin resistance [50].

Limitations of the study include the number of patients studied, especially men. In addition, up to 50% of the patients had received corticosteroids, chloroquine and/or methotrexate 1 month before entering the cohort, and these drugs are known to affect the lipid profile and eventually alter the true prevalence of MetS [46,51]. Our clinic is established in a tertiary care center, and there is a possibility of patient selection bias; patients with mild RA may especially be underrepresented. Also, our cohort of patients with early disease is limited to a population of Mexican Mestizos who have particular genetic background, socio-demographic characteristics, treatment availability and health system referral – all of these factors potentially affect both the disease itself and MetS; accordingly, our results may not be generalized. Owing to the limited years of follow-up of our cohort, the impact of MetS on CV outcomes was not assessed, and neither was the impact of individual components of MetS, which have been shown to perform better (than currently recommended MetS definitions) in identifying subclinical atherosclerosis in RA patients [52]. A limited number of comorbid conditions were assessed in our study (obesity, hypertension and diabetes) while others such as hyperuricemia and hypothyroidism that are recognized to be independent CV risk factors in the general population and in RA patients were not [53]. Finally, during 10-year follow-up there were up to 20% missing data and an imputation methodology was used.

## Conclusions

Mexican Mestizo RA patients referred to a tertiary care center had a prevalence of MetS variable upon the criteria applied, from 11 to 18%, but lower than matched, apparently healthy controls. Up to 9 years of follow-up, the MetS incidence rate was 34.5% and the annual MetS incidence rate decreased after the third year. Disease activity and MetS had a complex association favoring a vicious circle: greater cumulative disease activity and BMI were both predictors of incidental MetS, while incidental Mets and higher disease activity at baseline predicted worse outcomes. In such a clinical context, it appears that both MetS and disease activity need to be treated and effectively controlled in order to impact disease outcomes. Ultimately, CV risk will be also impacted.

## Abbreviations

ACCP: antibodies to cyclic citrullinated peptides
AHA/NHLBI: American Heart Association/National Heart, Lung and Blood Institute
BMI: body mass index
C-HDL: high-density lipoprotein-cholesterol
CRP: C-reactive protein
CV: cardiovascular
DAS28: disease activity score evaluated in 28 joints
DMARD: disease-modifying anti-rheumatic drug
ESR: erythrocyte sedimentation rate
GLU: glucose
MetS: metabolic syndrome
RA: rheumatoid arthritis
TG: triglycerides
